# Supplementary material for: An engineered variant of MECR reductase reveals indispensability of long-chain acyl-ACPs for mitochondrial respiration
Source: Nat Commun. 2023 Feb 4;14:619. doi: 10.1038/s41467-023-36358-7 (PMC9899272; doi:10.1038/s41467-023-36358-7)
Supplement: Supplementary file 1 — Supplementary Information [file 41467_2023_36358_MOESM1_ESM.pdf]

Supplementary information for

An engineered variant of MECR reductase reveals indispensability of long-chain acyl-ACPs for mitochondrial respiration

M. Tanvir Rahman[1], M. Kristian Koski[2], Joanna Panecka-Hofman[3,4], Werner Schmitz[5], Alexander J. Kastaniotis[1], Rebecca C. Wade[4,6], Rik K. Wierenga[1], J. Kalervo Hiltunen[1] and Kaija J. Autio[1]*

[1]*Faculty of Biochemistry and Molecular Medicine, University of Oulu, Oulu, Finland*

[2]*Biocenter Oulu, University of Oulu, Oulu, Finland*

[3]*Faculty of Physics, University of Warsaw, Warsaw, Poland*

[4]*Molecular and Cellular Modeling Group, Heidelberg Institute for Theoretical Studies (HITS), Heidelberg, Germany*

[5]*Faculty of Biochemistry and Molecular Biology, University of Würzburg, Würzburg, Germany*

[6]*Zentrum für Molekulare Biologie (ZMBH), DKFZ-ZMBH Alliance and Interdisciplinary Center for Scientific Computing (IWR), Heidelberg University, Heidelberg, Germany*

* corresponding author

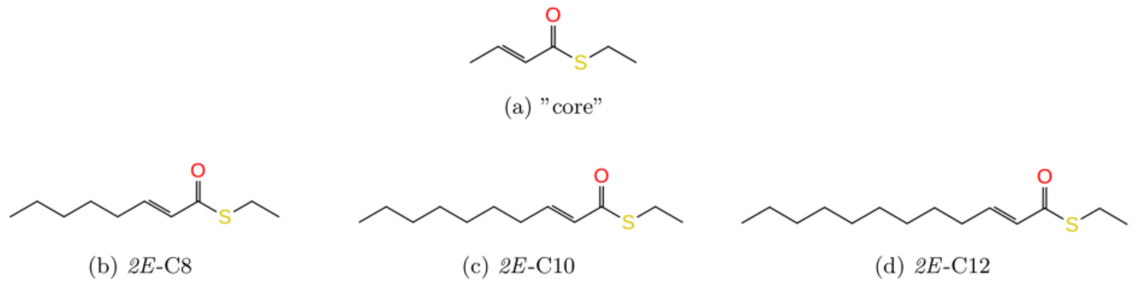

Supplementary figure 1. The substrate fragments docked to the MECR active site. (a) The substrate fragment – termed `core' elsewhere – which served as a reference in restrained docking simulations. (b) *2E*-C8, (c) *2E*-C10 and (d) *2E*-C12 substrate fragments docked to the MECR active site. Hydrogens are not shown.

Supplementary table 1. Single point mutations of G165 and their predicted effects based on modelling of the minimized MECR complexes with 2E-alkenoyl substrates. The higher number of stars, the more favorable mutation (subjective estimate based on the modelling and visual analysis). The mutations considered as the most promising are in bold font.

Mutation	Stars	Pros	Cons	Torsion scan
**G165H**	***	should block substrates >C9, possible favorable hydrogen bonds	may be too large (but it seems to fit)	probable rotamer should be effective
**G165L**	***	should block substrates >C8-C9	side chain may be too close to the Lys316 side chain	probable rotamer may be effective
**G165Q**	***	favorable interaction network if in the assumed conformation, should block substrates >C8/C9	the effect of mutations may depend on the conformation	slight inter-atomic clashes present
**G165F**	**	could block substrates >C8	more risky, less favorable in terms of interactions than mutation to His	a probable conformation that could be effective, but also some clashes (for which protein could however adapt)
**G165M**	**	should block substrates >C9	a risk of changed conformation	there are alternative, less effective, conformations (then blocking chains >C12)
**G165I**	**	should block substrates >C10	-	probable rotamer should have an effect
G165V	**	should block substrates >C10	may be too small	very frequent conformation (>70%) seems to be effective
G165N	**	a favorable interaction network (with Lys316 and C=O of Pro130); should block substrates >C10	-	a probable conformation could be effective
G165A	*	could block >C11-C12	should be combined with other mutations (too small alone)	-

Supplementary table 2. Single point mutations of I129 and their predicted effects based on modelling of the minimized MECR complexes with 2E-alkenoyl substrates. The higher number of stars, the more favorable mutation (subjective estimate based on modelling and visual analysis). The mutations considered as the most promising are in bold font.

Mutation	Stars	Pros	Cons	Torsion scan
I129M (exp.)	*	-	too far and too small to have an effect	-
**I129H**	***	could block >C10	favorable interactions for protonated His	effective rotamer is not very frequent (1%)
**I129F**	**	could block >C10, better than Tyr	possibly: too close contacts	effective rotamer is not very frequent (2%)
I129Y	*	-	the effect would depend on conformation	clashes and/or unfavorable contacts
I129R	*	-	too large	-
I129D	*	-	too small	-
I129E	*	-	unfavorable interactions	-
I129Q	*	-	unfavorable interactions	-

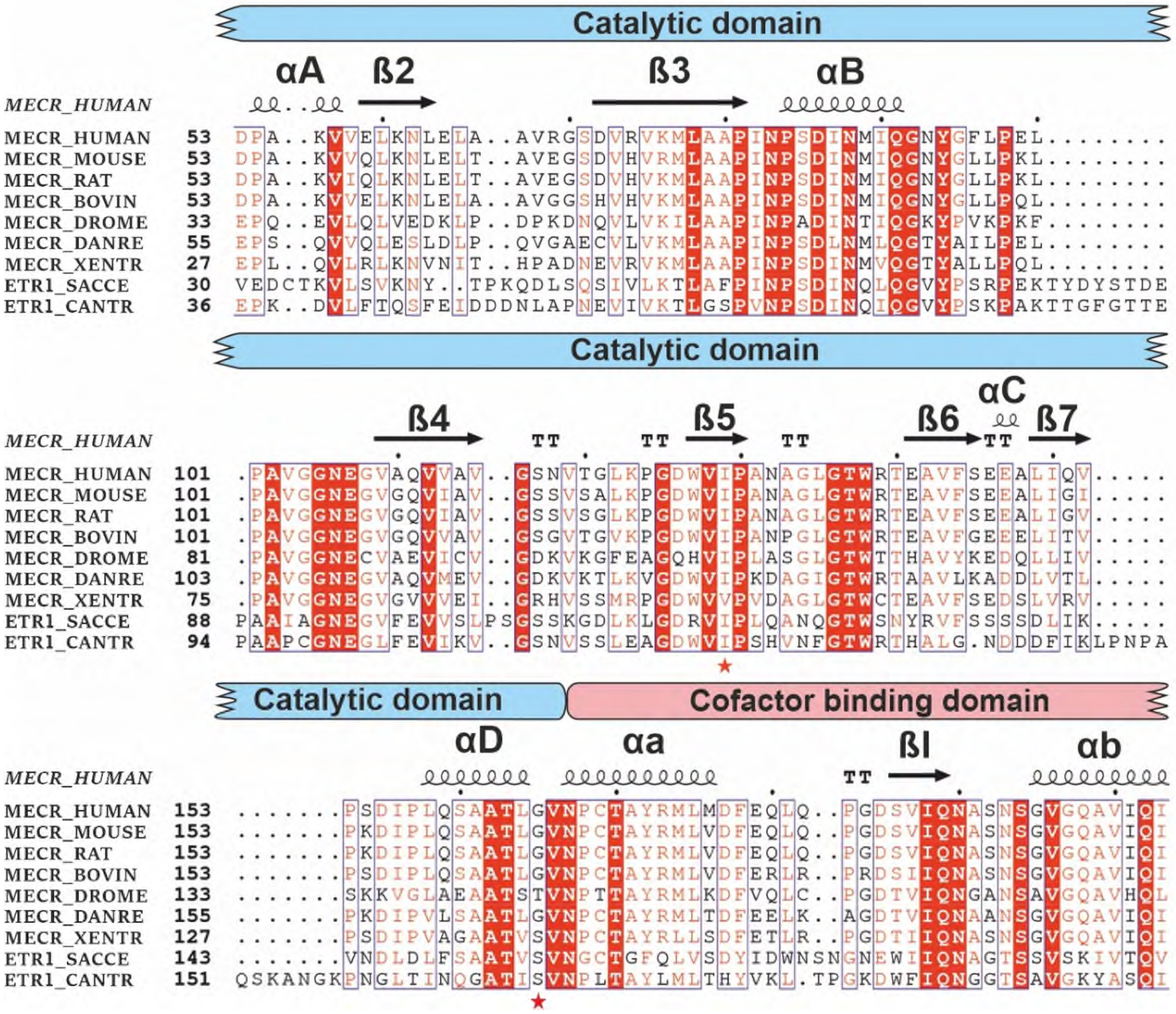

Supplementary figure 2. Multiple sequence alignment of mitochondrial 2E-enoyl reductases from various species. Amino acid residues from D53 to I203 of human enzyme (MECR_Human) were aligned with the orthologs from *Mus musculus* (MECR_Mouse), *Rattus norvegicus* (MECR_Rat), *Bos taurus* (MECR_Bovine), *Drosophila melanogaster* (MECR_Drome), *Danio rerio* (MECR_Danre), *Xenopus tropicalis* (MECR_Xentr), *Saccharomyces cerevisiae* (Etr1_Sacce) and *Candida tropicalis* (ETR1_Cantr). The secondary structures of the human enzyme are shown above the sequences and catalytic domain and cofactor binding domain are indicated above the secondary structure. Four last β-strands of the 7-stranded antiparallel β-sheet of the catalytic domain, and the first two α-helices and the first β-strand, βI, of the Rossmann fold of the cofactor binding domain are shown. I129 and G165 are marked with red asterisks under the sequence. The conserved residues are highlighted in red. The sequence alignment was prepared by using Clustal Omega[1]. The secondary structure elements and residue numbers corresponding to human MECR were generated by using ESPript 3.0[2]. The alignment of full length human MECR is published previously[3].

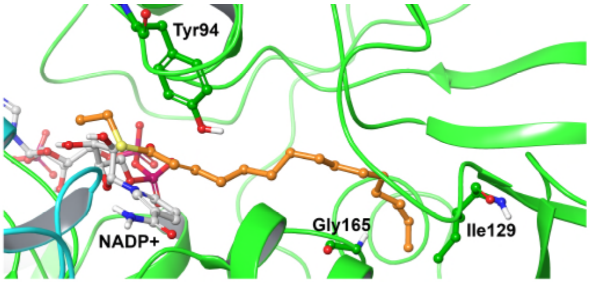

(a) WT

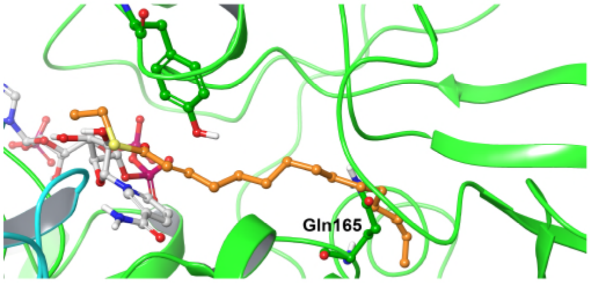

(b) G165Q

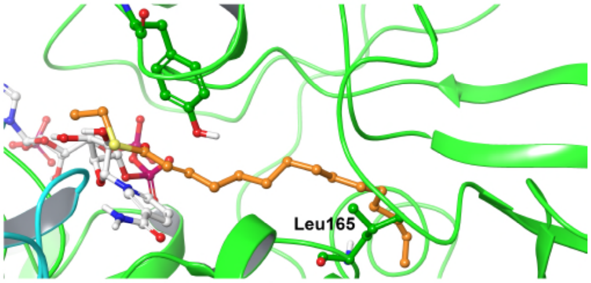

(c) G165L

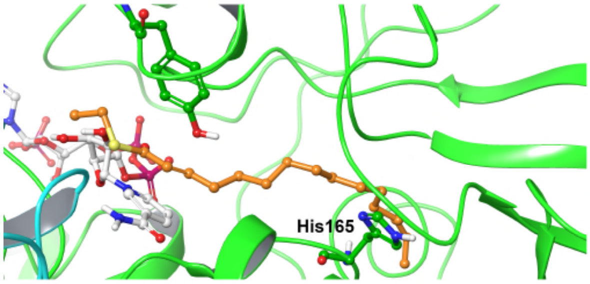

(d) G165H

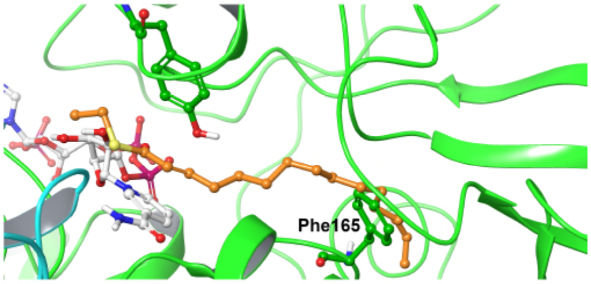

(e) G165F

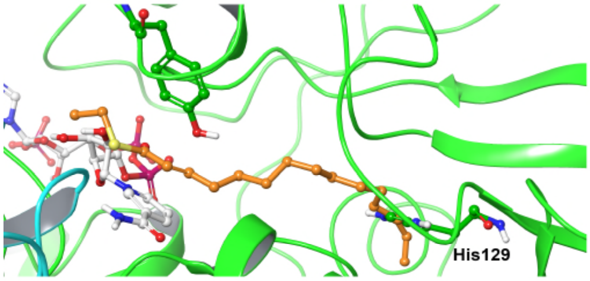

(f) I129H

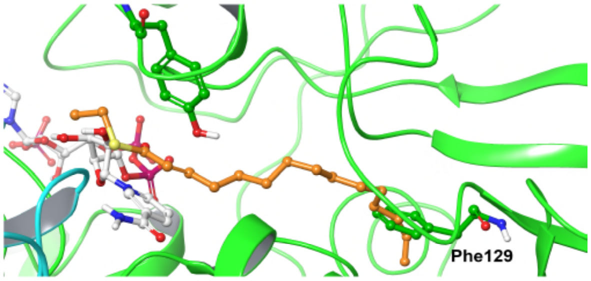

(g) I129F

Supplementary figure 3. **Designed mutants in the modeled complex of the wild-type MECR with 2E-16 substrate fragment:** (a) wild-type; the mutants: (b) G165Q, (c) G165L, (d) G165H, (e) G165F, (f) I129H, (g) I129F. The human wild-type MECR holoenzyme was modelled based on the crystal structure with PDB entry 2VCY; the cofactor $NADP^+$ (white carbons) and the C16 substrate fragment (orange carbons) were modelled-in based on the *C. tropicalis* ETR1 crystal structure (PDB entry 4WAS, which is the crystal structure of the Etr1p/$NADP^+$/crotonyl-CoA complex). The two mutated residues G165 and I129 and the catalytic residue Y94 are shown. The two subunits of the MECR enzyme are displayed in cartoon representation, colored green and cyan (A and B, respectively).

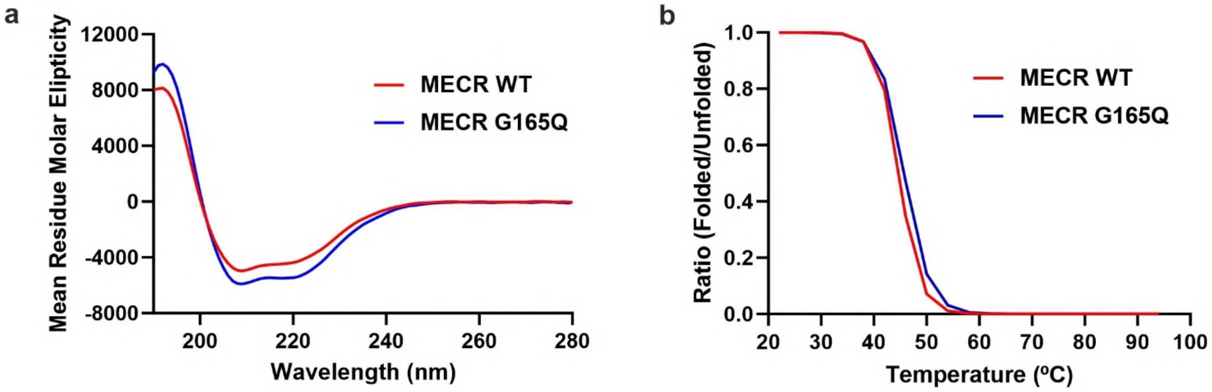

Supplementary figure 4. The secondary structure analysis and thermal stability measurement of MECR wild-type and G165Q variant. (a) CD spectra of MECR WT and MECR G165Q. Wild-type and mutant proteins show a typical α-helical profile with negative ellipticity at 222 nm and 208 nm and a positive ellipticity at 195 nm. (b) Thermal melting profile (at 222 nm) of wild-type MECR and mutant G165Q purified proteins. The CD melting curves show that the wild-type MECR protein has a T_m of 45.4°C and mutant G165Q protein has a T_m of 45.7°C. Source data are provided as a Source data file.

Supplementary table 3. Crystal data collection, data processing and refinement statistics

Data set	Wild-type MECR	MECR G165Q mutant
X-ray source	DLS, I04	DLS, I04
Data processing software	XDS/Aimless	DIALS/Aimless
**Data processing statistics**		
Unit cell parameters (A,°)	a=71.47<br>b=71.47<br>c=145.09<br>α= β = 90<br>γ = 120	a=71.52<br>b=71.52<br>c=143.77<br>α= β = 90<br>γ = 120
Space group	$P3_121$	$P3_121$
Resolution range (Å) (outer shell)	61.89-1.85<br>(1.90-1.85)	56.89-2.02<br>(2.07-2.02)
V_m (Å^3/D)	2.8	2.8
Number of subunits per asymmetric unit	1	1
Number of observations (outer shell)	722165 (41100)	563374 (41126)
Redundancy (outer shell)	19.3 (15.1)	19.6 (19.8)
Completeness (%) (outer shell)	100 (100)	100 (100)
I/s(I) (outer shell)	28.1 (1.5)	23.5 (1.3)
R_{merge} (%) (outer shell)	4.7 (189.4)	5.6 (247.5)
R_{pim} (%)(outer shell)	1.1 (49.7)	1.3 (56.8)
Wilson B factor (Å^2)	43.9	54.4
**Refinement statistics**		
Resolution (Å)	61.89-1.85 (1.90-1.85)	56.95-2.02 (2.07-2.02)
R_{work} (%)	18.91 (29.1)	21.8 (33.2)
R_{free} (%)	22.31 (35.8)	25.3 (34.5)
Number of reflections	34057 (2469)	25862 (1889)
Number of atoms[1]		
All	2886	2717
protein	2657	2655
$(PO_4)_2$ / $(SO_4)_2$	5	-
Cl	4	3
glycerol	12	-
acetate	8	-
waters	200	59
Rmsd bond length (Å)	0.009	0.005
Rmsd bond angle (°)	1.3	1.0
Average B factors (Å^2)		
all atoms	59.0	90.0
protein	59.0	90.1
$(PO_4)_2$ / $(SO_4)_2$	112.9	-
Cl	52.7	68.4
glycerol	68.1	-
acetate	73.8	-
waters	56.3	65.4
**Ramachandran plot**		
favored (%)	97.0	94.0
allowed (%)	3.0	5.0
outliers (%)	0.0	1.0
**PDB entry**	7AYB	7AYC

[1] Non-hydrogen atoms

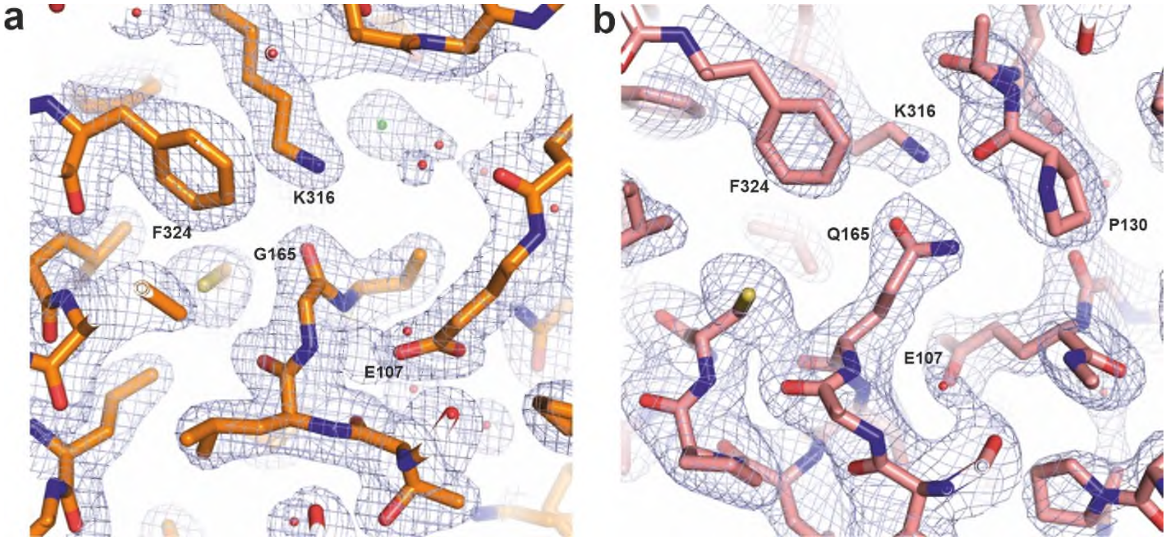

Supplementary figure 5. The 2Fo-Fc electron density map of the G/Q165 region of (a) the MECR wild-type centered around G165 and (b) the MECR G165Q variant centered around Q165. The counter level in both panels is 1.0 σ. Key amino acid residues are labelled. Water molecules and one chloride atom are shown in red and green spheres, respectively.

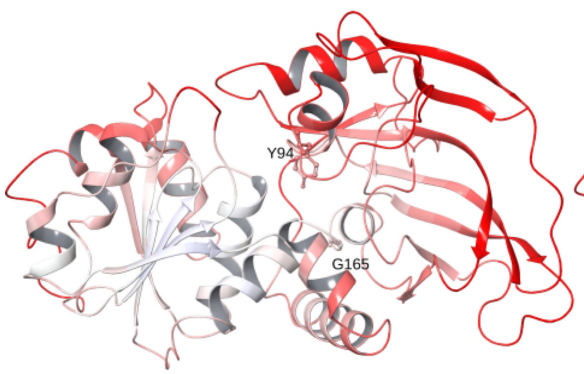

(a) WT

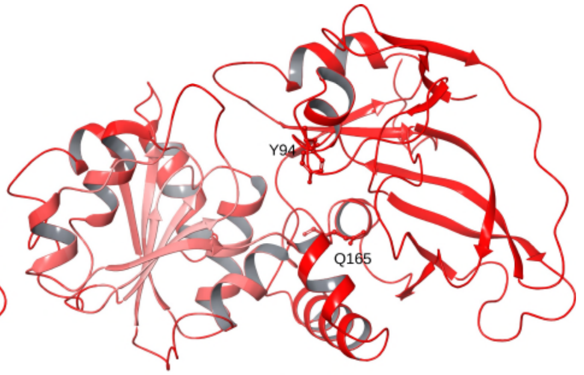

(b) G165Q

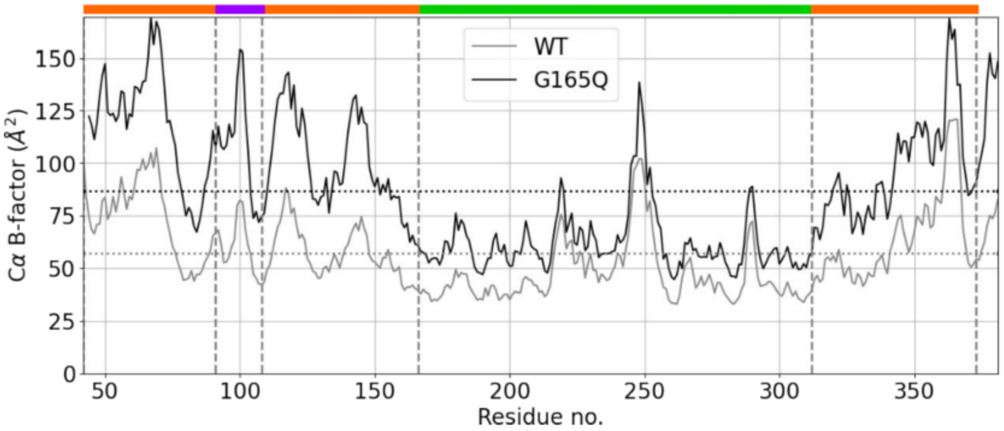

(c)

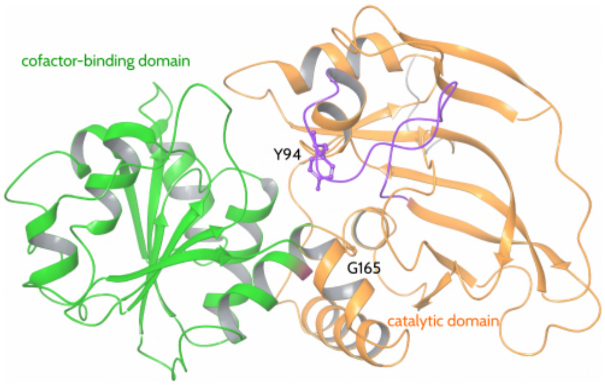

(d)

Supplementary figure 6. Comparison of the B factors of the crystal structures of wild-type and G165Q MECR. Crystal structures of the (a) wild-type (WT) and (b) G165Q MECR obtained in this work colored by B factor (red - high and white - low B factor). The catalytic residue Y94 and the mutated residue 165 are marked. (c) B factor plot. The B factor of the Cα atoms (on the vertical axis) are plotted as a function of residue number. Domains are marked above the plot according to the color coding in panel (d). Dashed vertical grid lines mark the boundaries of the domains and the active site loop near Y94. Dotted horizontal lines correspond to the mean Cα B factors for the WT and G165Q MECR crystals. (d) The wild-type MECR crystal structure colored by domains. The catalytic domain is shown in orange, the cofactor-binding domain in green and the loop 91-108 containing Y94 is shown in violet.

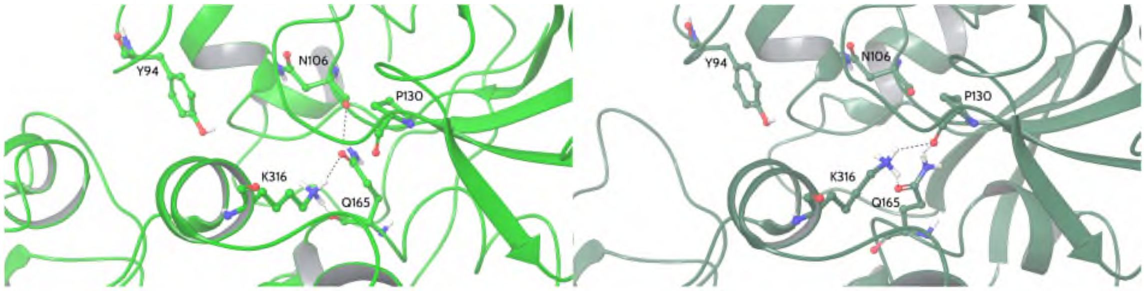

(a) model (b) crystal structure

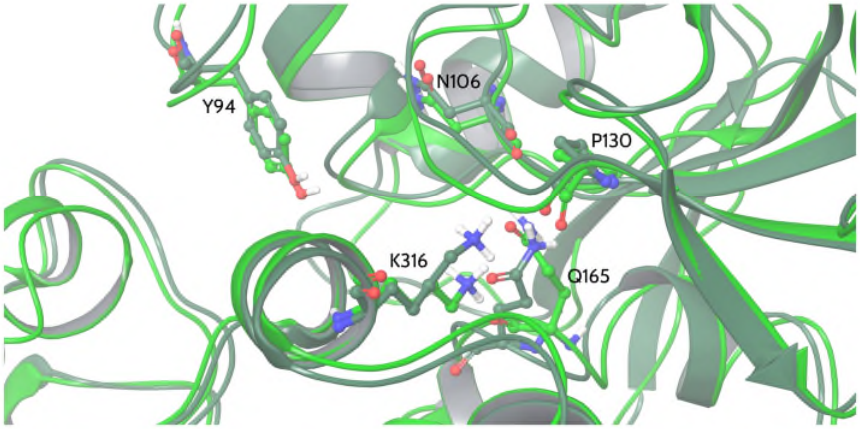

(c) superposed model and crystal structure

Supplementary figure 7. **The mutated residue Q165 and the residues that create hydrogen bonds with the Q165 side chain in the wild-type MECR model and in the crystal structure.** The mutated residue Q165 and the residues that create hydrogen bonds with the Q165 side chain in the wild-type MECR model (a) and in the crystal structure obtained in this work (b). The two structures superposed are shown in panel (c). Hydrogen bonds of the Q165 side chain are displayed as dashed lines in (a) and (b). Y94 is also shown as a reference. Hydrogens were added to the crystal structure for better visualization of hydrogen bonds. Hydrogen bonds were detected in Maestro 2017-4 with the following (default) criteria: max. distance 2.8 Å, donor min. angle 120.0^{o}, acceptor min. angle 90°.

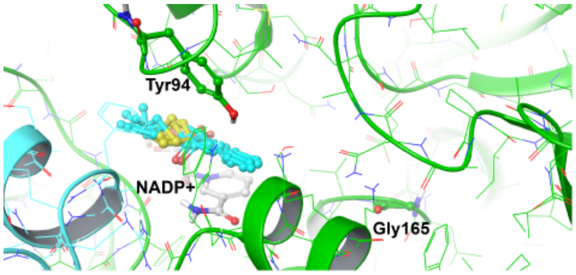

(a) "core"

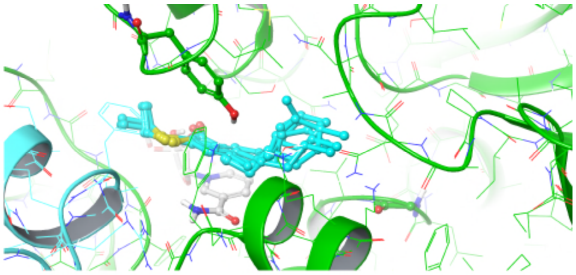

(b) *2E*-C8

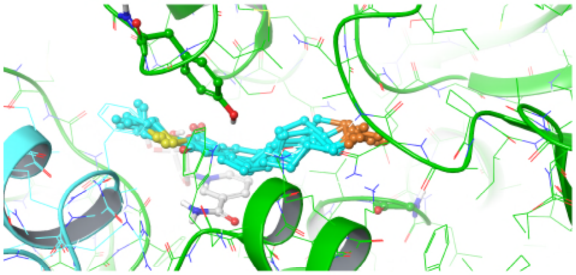

(c) *2E*-C10

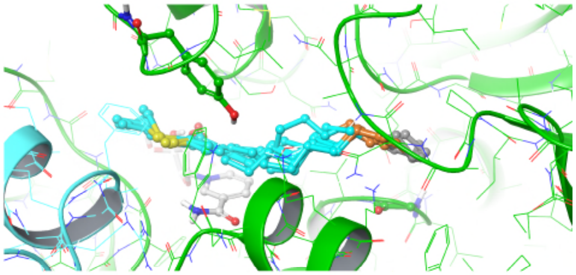

(d) *2E*-C12

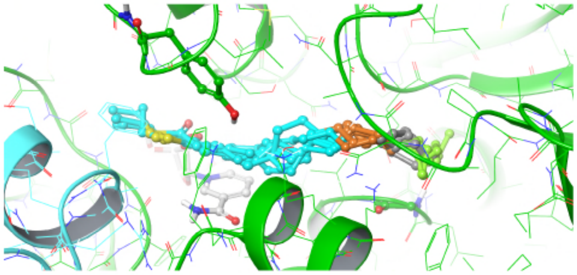

(e) *2E*-C14

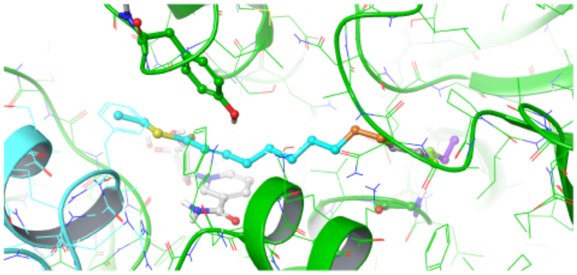

(f) *2E*-C16

Supplementary figure 8. The docking results of substrate fragments to the wild-type MECR crystal structure (PDB entry 7AYB): (a) the substrate core (with 2-enoyl moiety, see Supplementary figure 1a), (b) *2E*-C8, (c) *2E*-C10, (d) *2E*-C12, (e) *2E*-C14, (f) *2E*-C16. The poses were restrained to the initial position of substrate core with heavy-atom RMSD threshold of 2 Å. The obtained docking poses, additionally filtered by the core RMSD with threshold of 1.4 Å, are shown. The consecutive C doublets over C8 are differently colored (orange, gray, green and violet). The protein chains A and B (calculated by using the crystal symmetry) are colored green and cyan, respectively, NADP$^+$ is colored light gray. Residues Y94 and G165 are shown for reference in ball-and-stick representation (and labeled in panel a). The docking results are summarized in Supplementary table 4.

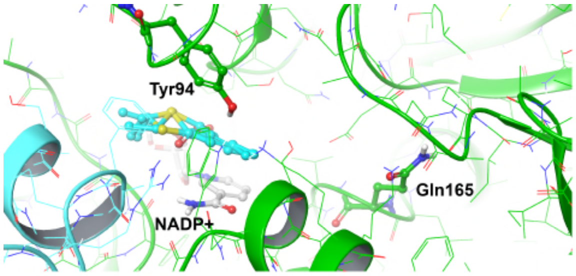

(a) "core"

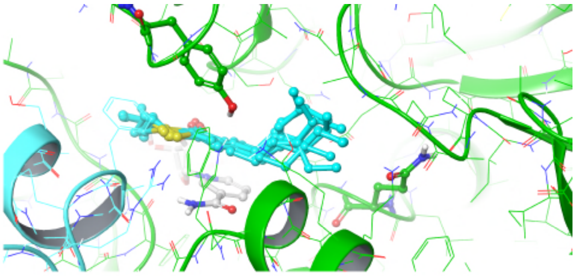

(b) *2E*-C8

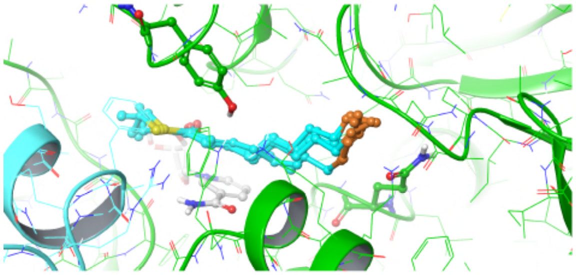

(c) *2E*-C10

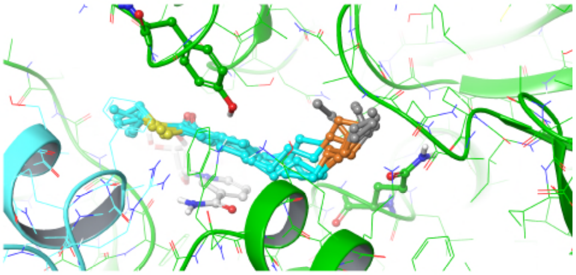

(d) *2E*-C12

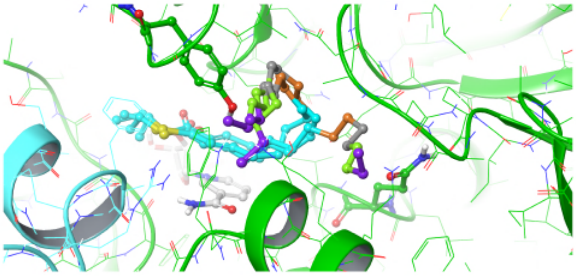

(e) *2E*-C16

Supplementary figure 9. The docking results of substrate fragments to the MECR G165Q crystal structure (PDB entry 7AYC): (a) the substrate core (with 2-enoyl moiety, see Supplementary figure 1a), (b) *2E*-C8, (c) *2E*-C10, (d) *2E*-C12, (e) *2E*-C16**.** The poses were constrained to the initial position of substrate core (with 2-enoyl moiety, Supplementary figure 1a) with heavy-atom RMSD threshold of 2 Å. The obtained docking poses, additionally filtered by the core RMSD with threshold 1.4 Å, are shown. 2E-C14 did not dock with the settings used. The consecutive C doublets over C8 are differently colored (orange, gray, green, violet). The protein chains A and B (calculated by using the crystal symmetry) are colored green and cyan, respectively, $NADP^+$ is colored light gray. Residues Y94 and the mutated Q165 are shown for reference in ball-and-stick representation (and labeled in panel a). The docking results are summarized in Supplementary table 4.

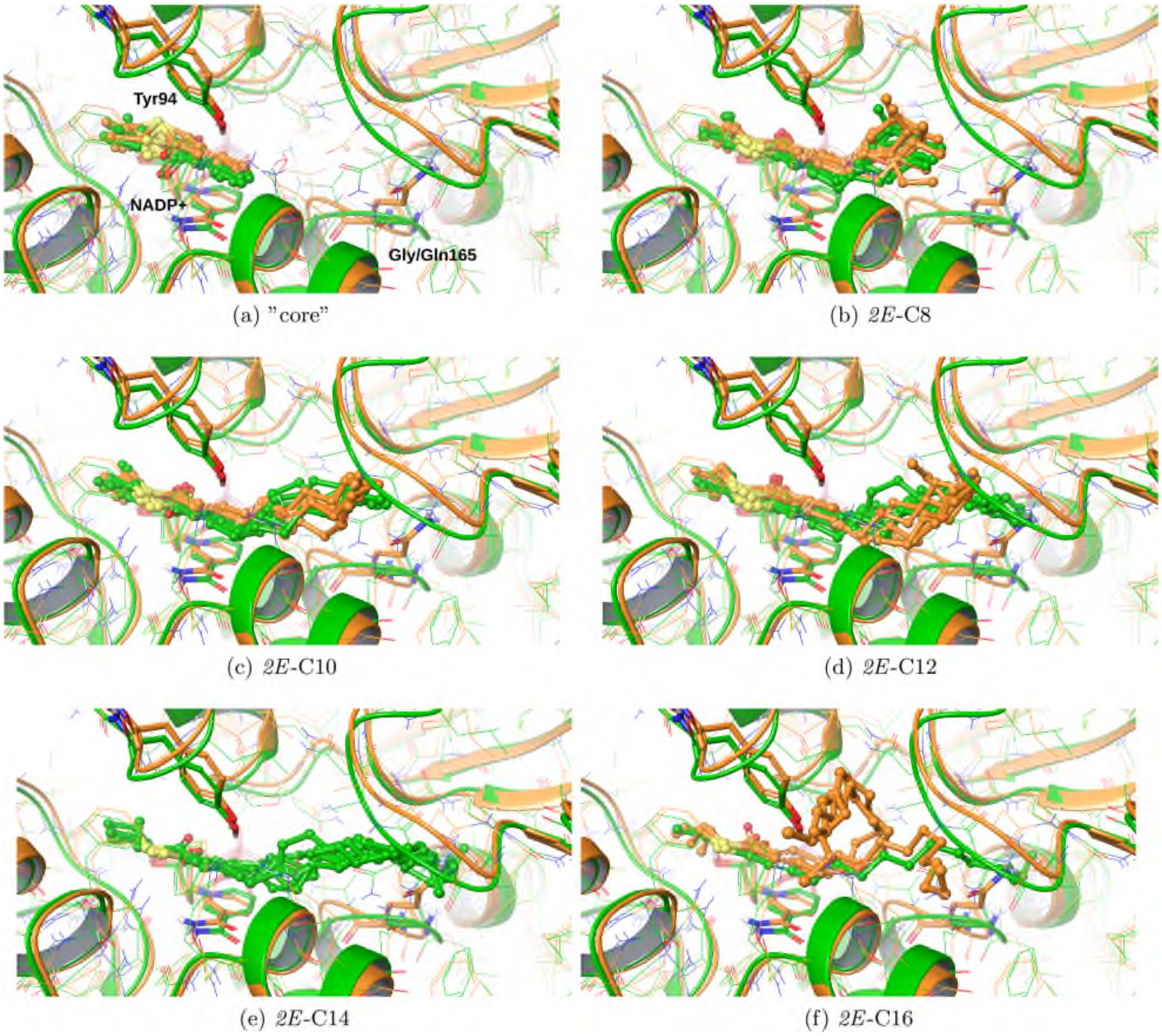

Supplementary figure 10. The aligned docking results of substrate fragments to the wild-type MECR and G165Q variant crystal structures: (a) the substrate core (with 2-enoyl moiety, see Supplementary figure 1a), (b) *2E*-C8, (c) *2E*-C10, (d) *2E*-C12, (e) *2E*-C14, (f) *2E*-C16. The aligned docking results of substrate fragments to the wild-type MECR are shown in green and G165Q variant are shown in orange. The crystal structures were obtained in this work. Y94 and G/Q165 are shown as sticks and labeled in panel (a). There were no docking solutions for *2E*-C14 in the G165Q variant (e). Non-polar hydrogens are not shown for clarity.

Supplementary table 4. The substrate "core" (2*E*-C4) and the 2*E*-C8–C16 substrate fragments docking results for the WT MECR and the G165Q MECR crystal structures obtained in this work. The docking results are filtered by RMSD of the 'core' fragment (Supplementary figure 1a) with threshold of 1.4 Å. Abbreviations: Ave - average, Std - standard deviation.

	**2*E*-C8**		**2*E*-C10**		**2*E*-C12**		**2*E*-C14**		**2*E*-C16**		**2*E*-C4 core**	
**no. of poses**												
**WT**	8		8		7		9		1		6	
**G165Q**	9		7		6		-		4		4	
**GScore (kcal/mol)**												
	**Ave**	**Std**	**Ave**	**Std**	**Ave**	**Std**	**Ave**	**Std**	**Ave**	**Std**	**Ave**	**Std**
**WT**	-1.9	0.3	-1.5	0.5	-1.4	0.3	-1.4	0.5	-1.8	-	-3.4	0.6
**G165Q**	-0.7	0.2	-0.4	0.2	0.1	0.3	-	-	0.1	0.4	-3.1	0.4
**van der Waals energy (kcal/mol)**												
	**Ave**	**Std**	**Ave**	**Std**	**Ave**	**Std**	**Ave**	**Std**	**Ave**	**Std**	**Ave**	**Std**
**WT**	-26.7	1.9	-28.9	2.3	-31.4	2.5	-27.0	5.5	-31.3	-	-19.2	2.2
**G165Q**	-27.1	0.9	-32.0	1.2	-25.0	3.0	-	-	-17.0	11.3	-19.4	1.9
**Internal ligand energy Eintern (kcal/mol)**												
	**Ave**	**Std**	**Ave**	**Std**	**Ave**	**Std**	**Ave**	**Std**	**Ave**	**Std**	**Ave**	**Std**
**WT**	4.5	2.5	5.9	2.6	6.5	3.0	8.9	3.2	6.2	-	1.4	1.4
**G165Q**	2.8	1.2	3.4	1.6	8.7	2.5	-	-	13.2	2.7	1.1	0.9
**core RMSD (Å)**												
	**Ave**	**Std**	**Ave**	**Std**	**Ave**	**Std**	**Ave**	**Std**	**Ave**	**Std**	**Ave**	**Std**
**WT**	0.7	0.2	0.8	0.3	0.6	0.1	0.8	0.2	0.7	-	1.0	0.4
**G165Q**	0.5	0.1	0.5	0.1	0.9	0.1	-	-	0.8	0.4	0.9	0.5

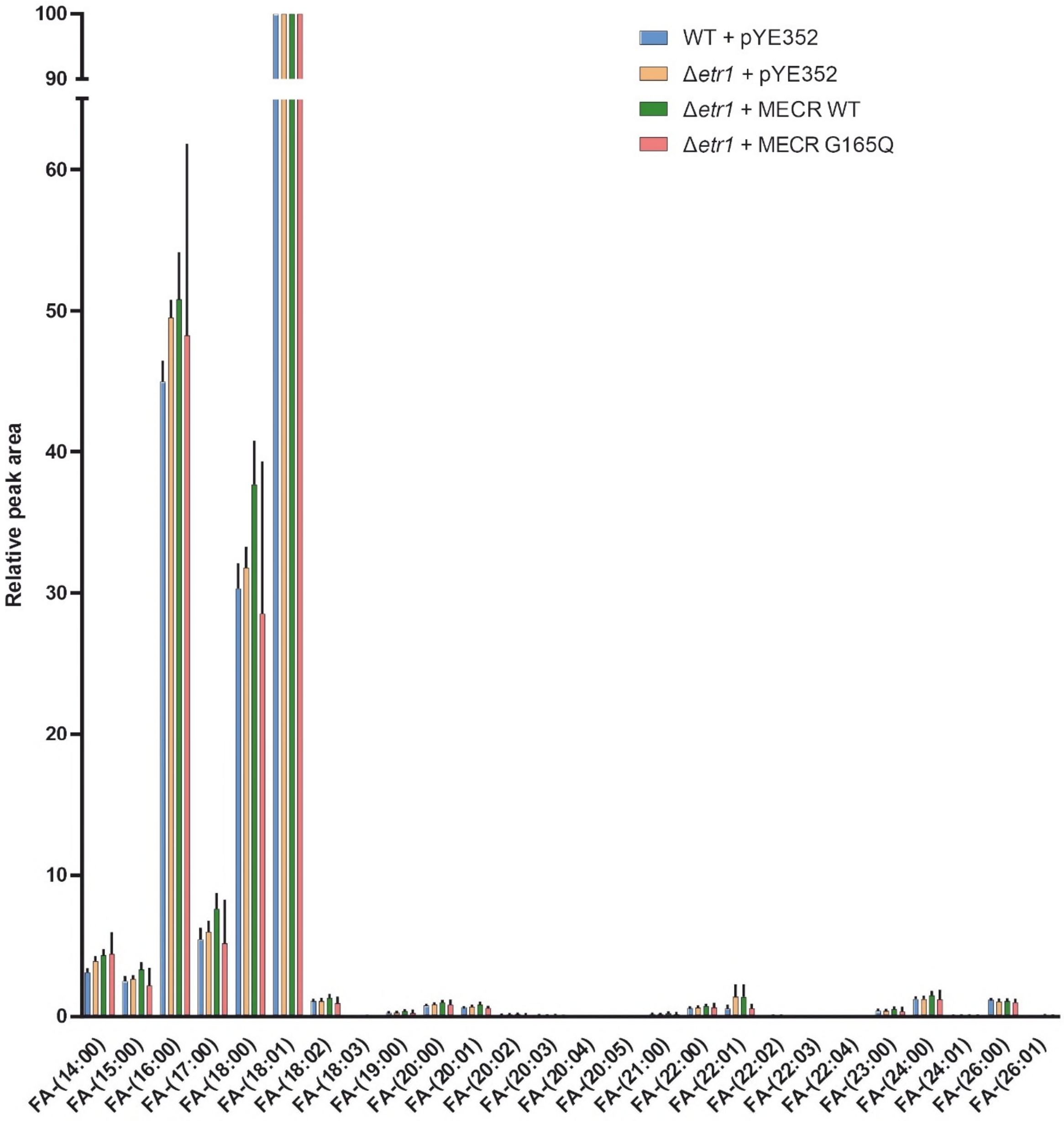

Supplementary figure 11. Total fatty acid profile from wild-type (WT), Δ*etr1* and Δ*etr1* expressing wild-type MECR or G165Q variant yeast cells. Source data are provided as a Source data file.

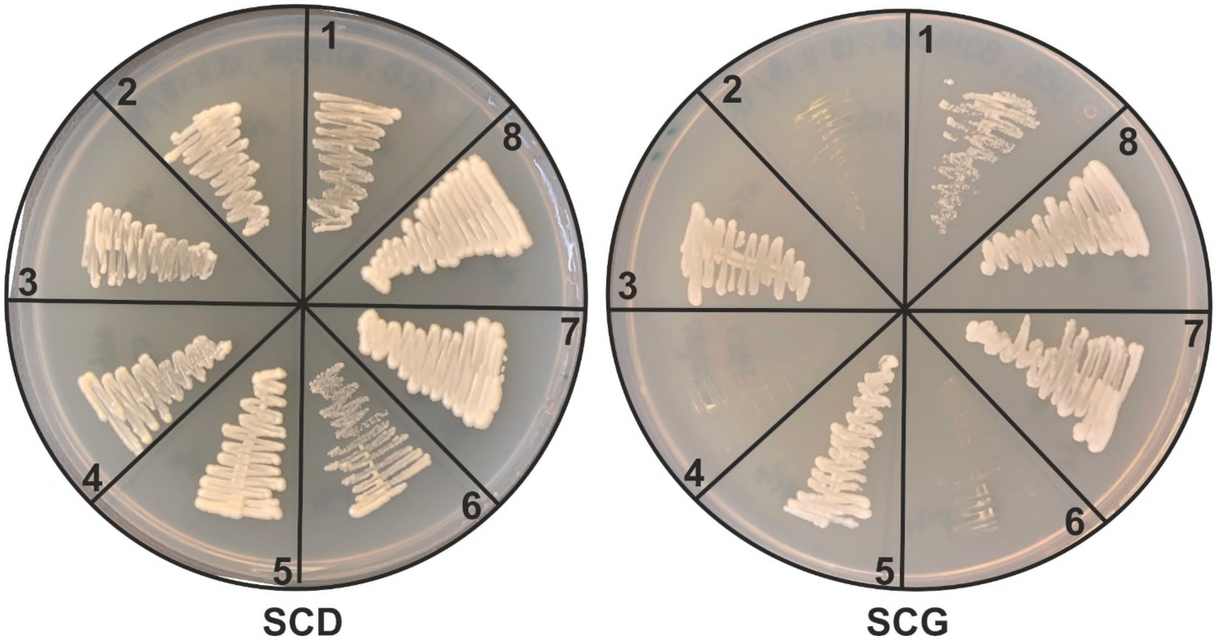

Supplementary figure 12. Respiratory growth assay to study potential loss of mitochondrial DNA. The respiratory growth of yeast was tested on non-fermentable SCG plate at +30°C for four days. Fermentable SCD plate was used as a control. The samples on the plates are: (1) wild-type Bj1991α, (2) Δ*etr1* Bj1991α, (3) wild-type Bj1991α transformed with empty pYE352 vector, (4) Δ*etr1* Bj1991α transformed with empty pYE352 vector, (5) Δ*etr1* transformed with wild-type MECR-pYE352 plasmid, (6) Δ*etr1* transformed with MECR mutant G165Q-pYE352 plasmid, (7) Δ*etr1* with MECR WT plasmid transformed with a plasmid expressing wild-type yeast Etr1p and (8) Δ*etr1* with G165Q plasmid transformed with a plasmid expressing wild-type yeast Etr1p.

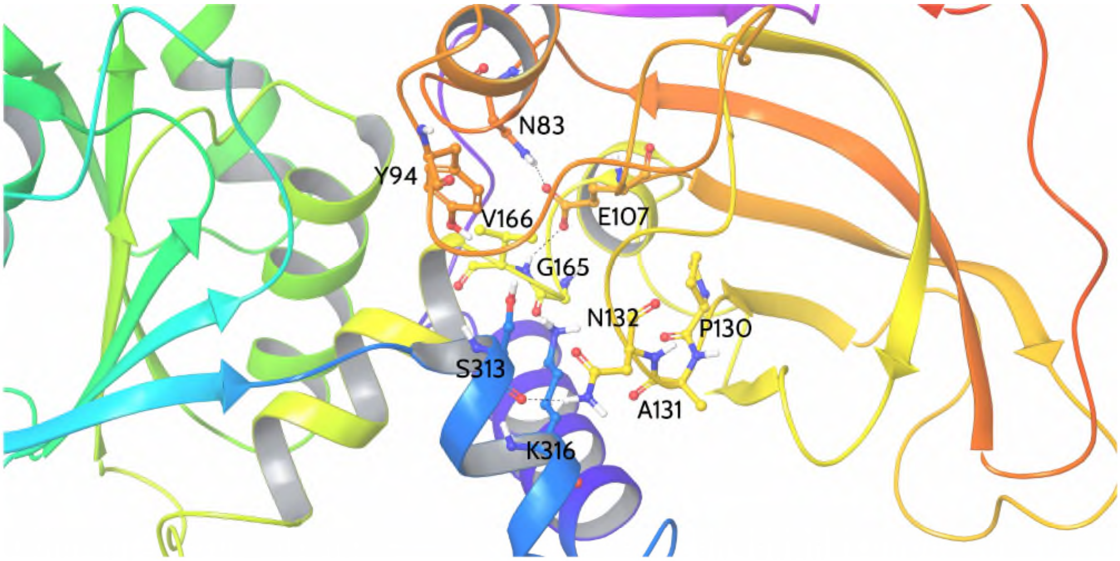

(a) WT

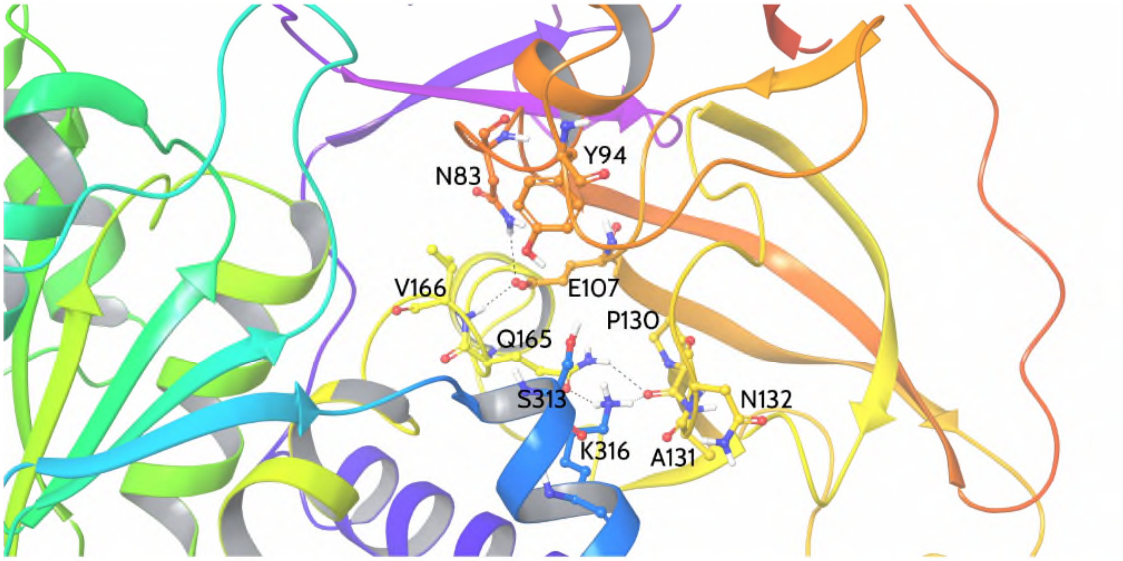

(b) G165Q

Supplementary figure 13. The residues with altered hydrogen-bonding network in the vicinity of active site in the crystal structures obtained in this work. (a) the WT MECR and (b) the G165Q MECR mutant. Y94 is also shown as a reference. Hydrogens were added to the crystal structure for better visualization of hydrogen bonds. The structures are colored by residue position. Hydrogen bonds created by Q165 in the mutant are shown in Supplementary figure 7. Hydrogen bonds were detected in Maestro 2017-4 with the following (default) criteria: max. distance 2.8 Å, donor min. angle 120.0^{o}, acceptor min. angle 90°.

Supplementary Table 5. Summary of the ConSurf webserver analysis results for selected residues. Amino acid occupancies are given for the most frequently occuring amino acids (occup. > 3%) at the most conserved positions (highlighted in yellow) and at the positions of mutations I129 and G165Q (highlighted in green).

3LATOM	SCORE	COLOR	CI	CI COLORS	MSA DATA	RESIDUE VARIETY
N83 : A	-1.398	9	-1.464, -1.366	9, 9	148/150	N (95%), H
Y94 : A	-1.446	9	-1.498, -1.412	9, 9	148/150	Y (100%)
E107 : A	-1.450	9	-1.498, -1.464	9, 9	150/150	K, E (99%)
I129 : A	-0.252	6	-0.429, -0.156	6, 5	150/150	R, I (60%), V (21%), M, T, A, C, L (11%), F
P130 : A	-0.526	7	-0.713,-0.429	7,6	150/150	M, A, H, S, Y, I, V, F, P, L
A131 : A	1.116	2	0.570, 1.132	3, 2	132/150	K, V, A, M, L, G, C, Q, F, Y, I, R, T, S, D, N
N132 : A	1.276	1	0.810, 1.623	3, 1	148/150	I, Y, R, S, T, N, D, K, V, E, H, A, M, L, G, Q, P, F
A133 : A	0.534	3	0.214, 0.810	4, 3	150/150	T, S, I, Y, R, D, N, A, M, K, V, E, P, F, L, Q
G165 : A	0.169	4	-0.156, 0.376	5, 4	150/150	A (9%), S (28%), T (25%), M, R, K, Y, V, I, F, N, G (14%), L, Q (5%)
V166 : A	-1.088	8	-1.185, -1.044	9, 8	150/150	I (8%), V (87%), L (3%), S, T, A
W311 : A	-1.244	9	-1.366, -1.185	9, 9	148/150	A, W (98%), M, N
S313 : A	-0.740	7	-0.888, -0.648	8, 7	147/150	V, A (7%), T (33%), S (45%), Q (7%), G, L, D, P, N, X
K316 :A	0.494	3	0.214, 0.570	4, 3	149/150	L, G, N, F, I, R, Y, V, K, A, S, T, M

3LATOM: The ATOM derived sequence in three letter code, including AA's positions
SCORE: The normalized conservation scores
COLOR: The color scale representing the conservation scores (9 – conserved, 1 – variable)
CONFIDENCE INTERVAL (CI): When using the Bayesian method for calculating rates, a confidence interval is assigned to each of the inferred evolutionary conservation scores.
CONFIDENCE INTERVAL COLORS: When using the Bayesian method for calculating rates, the color scale representing the lower and upper bounds of the confidence interval.
MSA DATA: The number of aligned sequences having an amino acid (non-gapped) from the overall number of sequences at each position
RESIDUE VARIETY: The residues variety at each position of the multiple sequence alignment

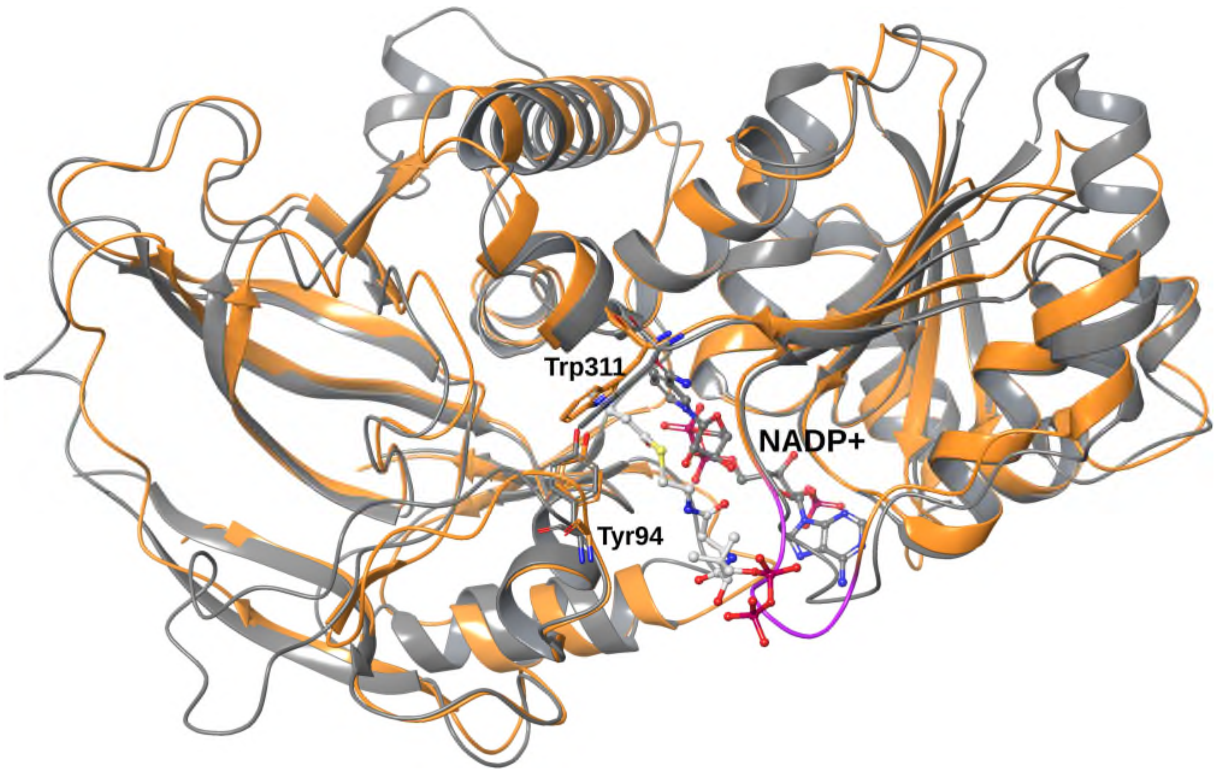

Supplementary figure 14. The aligned structures of human MECR (PDB entry 2VCY, in orange) and *C. tropicalis* Etr1 (PDB entry 4WAS, in gray). The $NADP^+$ cofactor and the fragment of coenzyme A are shown in ball-and-stick representation. The loop 287-291 of MECR, which has a distinctly differing conformation with respect to the *C. tropicalis* Etr1 loop, is shown in purple.

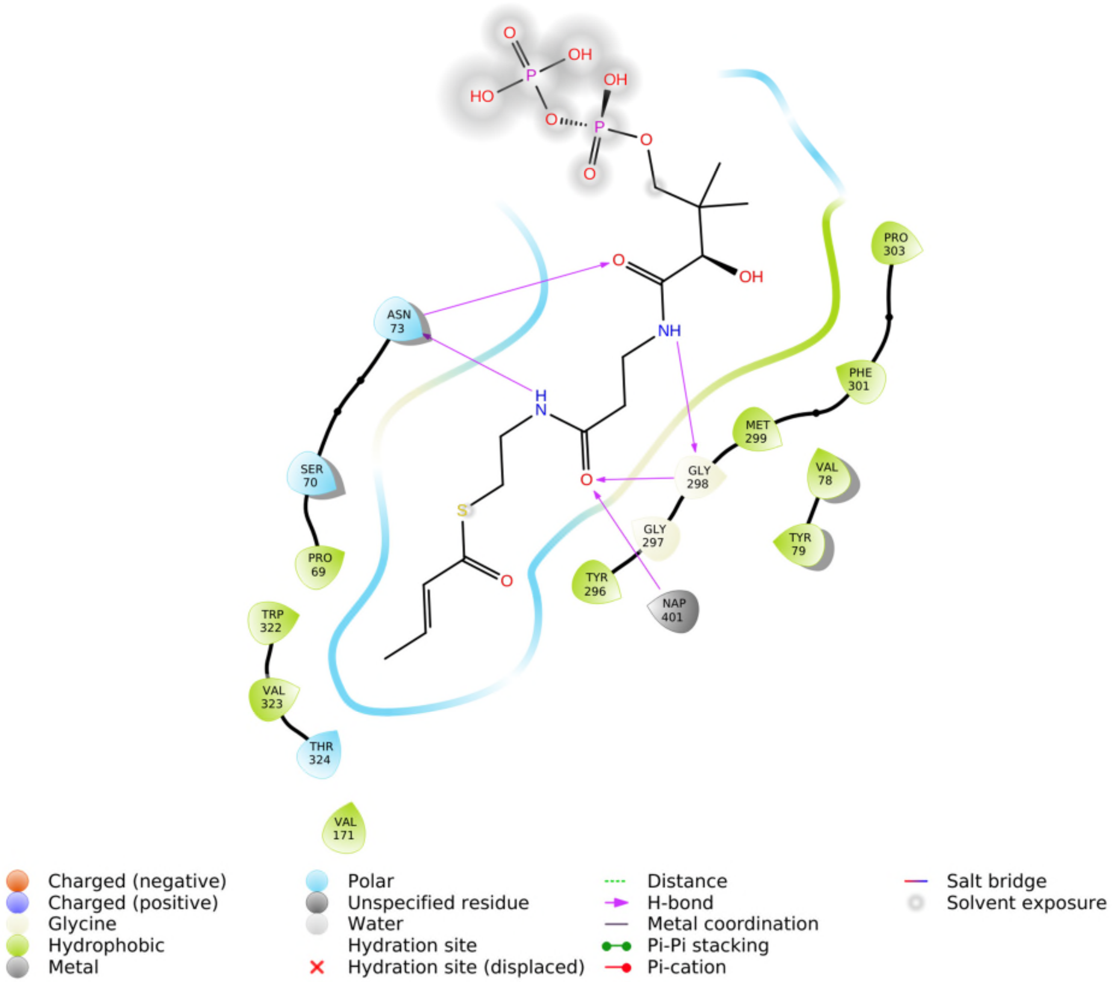

Supplementary figure 15. The hydrogen bonds of the coenzyme A fragment in the *C. tropicalis* Etr1 structure (PDB entry 4WAS, chain A). In order to visualize hydrogen bonds, hydrogens were added and minimized using the Maestro software suite ver. 2018-1.

Supplementary table 6. Primers used for this study

Primers	Sequence (5’ 3’)
GGT to CAT (Glycine to Histidine) _F	CTT CAG AGC GCT GCC ACC CTG CAT GTC AAT CCC TGC ACA GCC TAC
GGT to CAT (Glycine to Histidine) _R	GTA GGC TGT GCA GGG ATT GAC ATG CAG GGT GGC AGC GCT CTG AAG
GGT to CTT (Glycine to Leucine) _F	CTT CAG AGC GCT GCC ACC CTG CTT GTC AAT CCC TGC ACA GCC TAC
GGT to CTT (Glycine to Leucine) _R	GTA GGC TGT GCA GGG ATT GAC AAG CAG GGT GGC AGC GCT CTG AAG
GGT to CAA/CAG (Glycine to Glutamine) _F	CTT CAG AGC GCT GCC ACC CTG CAG GTC AAT CCC TGC ACA GCC TAC
GGT to CAA/CAG (Glycine to Glutamine) _R	GTA GGC TGT GCA GGG ATT GAC CTG CAG GGT GGC AGC GCT CTG AAG
GGT to TTT (Glycine to Phenylalanine) _F	CTT CAG AGC GCT GCC ACC CTG TTT GTC AAT CCC TGC ACA GCC TAC
GGT to TTT (Glycine to Phenylalanine) _R	CCT GTA GGC TGT GCA GGG ATT GAC AAA CAG GGT GGC AGC GCT CTG AAG
ATT to CAT (Isoleucine to Histidine) _F	CTG AAG CCA GGA GAC TGG GTG CAT CCA GCA AAT GCT GGT TTA GGA
ATT to CAT (Isoleucine to Histidine) _R	TCC TAA ACC AGC ATT TGC TGG ATG CAC CCA GTC TCC TGG CTT CAG
ATT to TTT (Isoleucine to Phenylalanine) _F	CTG AAG CCA GGA GAC TGG GTG TTT CCA GCA AAT GCT GGT TTA GGA
ATT to TTT (Isoleucine to Phenylalanine) _R	TCC TAA ACC AGC ATT TGC TGG AAA CAC CCA GTC TCC TGG CTT CAG
Forward sequencing primer	CACTGTAATGTCCAAATCG
Reverse sequencing primer	CTGAATTCATGAGTATGATC

References for supplementary info:

1. Chojnacki, S., Cowley, A., Lee, J., Foix, A. & Lopez, R. Programmatic access to bioinformatics tools from EMBL-EBI update: 2017. *Nucleic Acids Research* **45**, W550–W553 (2017).

2. Robert, X. & Gouet, P. Deciphering key features in protein structures with the new ENDscript server. *Nucleic Acids Research* **42**, (2014).

3. Chen, Z. J. *et al.* Structural enzymological studies of 2-enoyl thioester reductase of the human mitochondrial FAS II pathway: new insights into its substrate recognition properties. *Journal of Molecular Biology* **379**, 830–844 (2008).
